# Deathly Drool: Evolutionary and Ecological Basis of Septic Bacteria in Komodo Dragon Mouths

**DOI:** 10.1371/journal.pone.0011097

**Published:** 2010-06-21

**Authors:** J. J. Bull, Tim S. Jessop, Marvin Whiteley

**Affiliations:** 1 Section of Integrative Biology, University of Texas, Austin, Texas, United States of America; 2 Section of Molecular Genetics and Microbiology, University of Texas, Austin, Texas, United States of America; 3 Institute for Cellular and Molecular Biology, University of Texas, Austin, Texas, United States of America; 4 Center for Computational Biology and Bioinformatics, University of Texas, Austin, Texas, United States of America; 5 Department of Zoology, University of Melbourne, Melbourne, Australia; University of Oxford, United Kingdom

## Abstract

Komodo dragons, the world's largest lizard, dispatch their large ungulate prey by biting and tearing flesh. If a prey escapes, oral bacteria inoculated into the wound reputedly induce a sepsis that augments later prey capture by the same or other lizards. However, the ecological and evolutionary basis of sepsis in Komodo prey acquisition is controversial. Two models have been proposed. The “bacteria as venom” model postulates that the oral flora directly benefits the lizard in prey capture irrespective of any benefit to the bacteria. The “passive acquisition” model is that the oral flora of lizards reflects the bacteria found in carrion and sick prey, with no relevance to the ability to induce sepsis in subsequent prey. A third model is proposed and analyzed here, the “lizard-lizard epidemic” model. In this model, bacteria are spread indirectly from one lizard mouth to another. Prey escaping an initial attack act as vectors in infecting new lizards. This model requires specific life history characteristics and ways to refute the model based on these characteristics are proposed and tested. Dragon life histories (some details of which are reported here) prove remarkably consistent with the model, especially that multiple, unrelated lizards feed communally on large carcasses and that escaping, wounded prey are ultimately fed on by other lizards. The identities and evolutionary histories of bacteria in the oral flora may yield the most useful additional insights for further testing the epidemic model and can now be obtained with new technologies.

## Introduction

The Komodo dragon (*Varanus komodoensis*) is the world's largest lizard, with a mass up to 90 kg and a length of 3 m. It is restricted to five small islands in Eastern Indonesia [1]. Here, it is an apex predator, with adult lizards killing the largest ungulate prey found on the island–water buffalo, pigs and Timor deer–that often equal or exceed its own body mass [2]. Individual dragons often kill their prey directly but also feed on carcasses of prey killed by other lizards or other agents. A large carcass enables multiple dragons to feed on one carcass at the same time.

In some cases, the ultimate demise of a prey is purportedly due to more than just direct bite induced trauma, involving bacterial sepsis acquired from the lizard's bite [2], [3] or envenomation [4]. Whilst direct bite inflicted injury is the most intuitive and oft observed mechanism to rapidly dispatch prey, the role of bacteria or venom to aid prey death is poorly known. In a study of multiple lizards, 58 species of bacteria were identified from the saliva and oral cavities [3], 93% of which are classified as potentially pathogenic [5]. At least one species, tentatively identified as *Pastuerella multocida*, caused high mortality among mice injected with Komodo dragon saliva [3]. Thus, the potential exists for bite-induced sepsis to contribute to prey mortality, but the bacteria may instead be coincidental to any effect on prey [4].

Here we focus on the possible origins and ecological bases of sepsis-inducing bacteria in the mouths of komodo dragons. Auffenberg [2] proposed that the bacteria were beneficial to the lizards, in essence a slow-acting venom that facilitated prey capture by the attacking lizard or other lizards (termed here the ‘bacteria as venom’ model). He offered no mechanism by which the bacteria were acquired by lizards, however. Fry et al. [4] questioned this interpretation, and proposed that sepsis-inducing bacteria were more plausibly acquired passively from prey and other environmental sources, with no role in prey acquisition (the ‘passive acquisition’ model). That model fits the observation that captive lizards (and presumably newborns) lack sepsis-inducing bacteria [3].

The purpose of this paper is to propose and analyze a third model, the ‘lizard-lizard epidemic’ model. Instead of interpreting the infectious oral flora of dragons as either beneficial to the lizards or as a byproduct of feeding on mammals and carrion, the model proposes that bacteria spread epidemically among lizard mouths via prey that escape an initial attack. Escaping, infected prey thus serve as vectors to spread the infection among lizard mouths. Predictions are developed for this model and compared to Komodo dragon life history details in attempting to refute the model and discriminate it from the alternatives. Original observations on wild dragons are reported here as part of this test.

## Results

### The lizard-lizard epidemic model

The model describes the spread of a bacterium among lizard mouths. In humans and other highly social species, an oral infection might be spread directly by kissing or by other personal contact that led to contamination of the mouth. Like most lizards, Komodo dragons are largely solitary from birth and generally remain asocial [6], with no evidence for behaviors that would enable viable rates of direct bacterial transmission between individuals' mouths [2]. Hence direct spread seems unlikely, but prey surviving an initial attack that acquired the bacteria and developed a sepsis could act as a vector in the indirect bacterial spread to other lizards if other lizards ate those infected prey. The model is thus that a lizard infects a prey, and that prey in turn infects the mouths of other lizards.

The oral bacterium causes sepsis. Sepsis has important effects on transmission both from lizard to prey and from prey back to lizard. First, a sepsis-causing, oral bacterium may be established in the mammalian prey easily by a lizard bite, because the bite initiates an infection. Auffenberg [2] noted that dragon teeth were adept at cutting and tearing flesh, an attribute that would easily establish an infection. Second, sepsis in the prey doubly facilitates transmission to a new lizard: sepsis weakens the prey, rendering it prone to eventual capture, and sepsis causes high bacterial densities in the prey, enhancing colonization of the mouth of any lizard eating the tissues. The one complication in this scenario is that a time lag is required between the two steps. A bitten prey requires hours or days to develop sepsis, and then only if it is alive. The prey must therefore survive the initial attack long enough to develop sepsis but then be eaten by lizards.

We now develop formal predictions from this model. A useful concept of an infectious agent (‘disease’) is the basic reproductive number, R_0_, defined as the average number of new infections transmitted by the first infected individual in a population of naive hosts. R_0_ applies over the lifetime of the first infection, so it is, in essence, offspring number of a disease agent. R_0_ must exceed 1.0 for the disease to spread and be maintained; values progressively larger than 1.0 result in faster spread and higher equilibrium densities of infected hosts. (We use the term ‘disease’ without prejudice for whether it harms the lizard.) With this requirement, the first lizard to acquire the sepsis-inducing oral flora must on average spread it to more than one other lizard before the first lizard dies or loses the flora.

### Main properties of the lizard-lizard epidemic model

Under this model, the ecological characteristics fostering the spread of a sepsis-inducing oral flora include the following:

1. Prey escape lizard attacks after being bitten
2. Bacteria acquired from the lizard mouth cause a systemic infection in prey, achieving high densities in tissues that are normally consumed by lizards.
3. Infected prey are later eaten by lizards, enhanced by
  - The bacteria weaken or kill large prey, facilitating subsequent capture by lizards
  - Infected, large prey do not escape to habitats not frequented by dragons (e.g., savanna grassland, the majority of island habitat).
  - Prey are sufficiently large to enable multiple (unrelated) lizards to consume a single carcass
4. Bacteria survive in a dead carcass, colonize and reproduce in a lizard mouth.

The main consequence of these properties/assumptions is that the same infectious bacteria come to exist in different lizard mouths. Given that large prey carcasses are typically eaten by the largest lizards, the epidemic spread of infectious bacteria is largely confined to large lizards.

These characteristics are presented as qualitative requirements; there are necessarily quantitative constraints on them as well. The Methodsection provides a mathematical analysis of the problem using a standard ‘SI’ model (with categories of ‘susceptible’ and ‘infected’). Results of that analysis support these intuitive points and provide some interesting insights to the quantitative nature of parameter values required. However, at this point, there are no quantitative data for parameterizing such a model, so we limit the body of the paper to qualitative arguments.

### Dragon life histories and feeding ecology match requirements 1 and 3

We discuss these four points in order of the evidence we have to address them. The third point will be discussed before the second.

#### 1. Prey sometimes escape attacks with injuries

**Figure 1:**
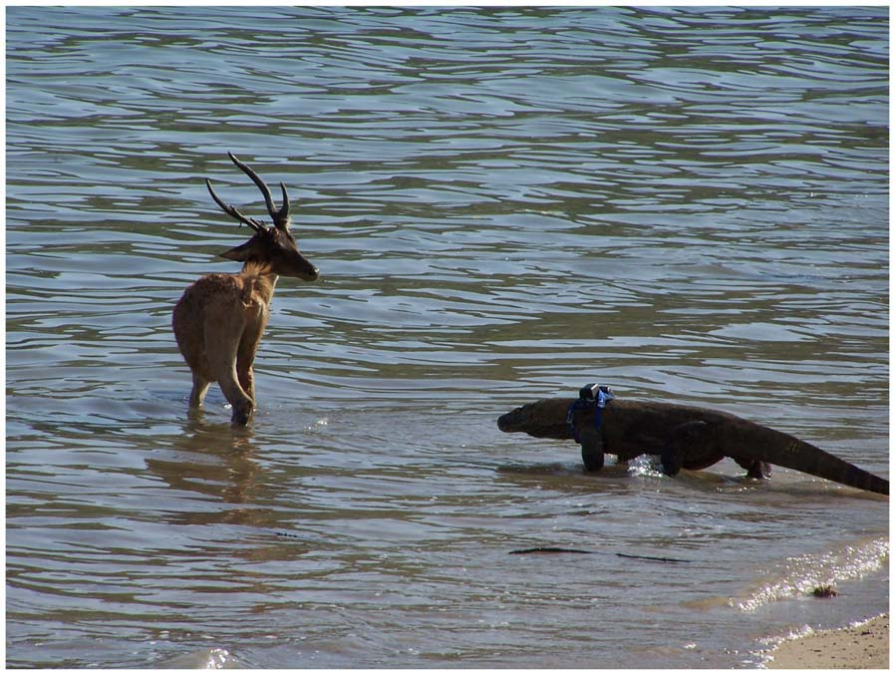
Pursuit of an escaped prey by a second dragon. An injured Timor deer that has escaped an initial lizard has fled to the ocean and is being stalked by a different dragon. This second lizard succeeded in killing the deer. The Komodo dragon pictured is wearing a GPS collar used for tracking the animal. Occasional prey escape is essential to the operation of the lizard-lizard epidemic model. (Photo by Achmad Ariefiandy.)

Auffenberg [2] reported that injured and wound-infected mammals (deer, mostly) were observed, uncommonly, but often enough to indicate that prey sometimes escaped attacks. During 2002–2009, TSJ (Tim Jessop) recorded 17 observations of lizard attacks on large prey, deer and pigs: 12 were successful (fatal) and 5 (30%) were unsuccessful (4 deer and 1 pig). When prey escaped the initial attack, they sustained bite injuries that included lacerations to limbs and rump. An escaped prey can subsequently recover, be killed soon in a second attack by other lizards, or succumb to its wounds and/or infection hours or days later. Of these 5 escapes, one animal was soon attacked and killed by a second lizard (Fig. 1), two died within hours without further attack, one was pursued by 4 other lizards as it fled, and one limped away without pursuit by other lizards. Thus 1–2 of 17 attacks resulted in prey that may have survived long enough to become infected.

It is perhaps no coincidence that prey escape rates are moderately high and that those prey are ungulate mammals as large as or larger than the lizards. Large prey size is likely key to the operation of this model: it (a) confers an increased probability of escape, (b) enables prey to be more tolerant of injury, enabling bacterial sepsis to develop and (c) imposes consumption limits on an individual lizard, so that multiple lizards feed concurrently or sequentially; an infected, large prey can potentially infect over a dozen lizards (see below).

#### 2. Prey are eaten by multiple lizards

Prey consumption by ‘other’ lizards (those not infecting the prey) is critical to the model. If injured prey are never encountered again or are only eaten by the lizard responsible for the initial attack, an oral infection would not spread. Perhaps surprisingly, dragon feeding behaviors are especially conducive to the spread of bacteria under this model. On a qualitative level, lizard density, movements and spatial structuring are sufficient and overlapping at least in some parts of the islands, that sick prey are likely to be consumed by one or more large lizards. The lizards scavenge carcasses (with a preference for fresh kills) and typically aggregate at kills of large prey (e.g., water buffalo, personal observations by TSJ and [2]). In the case of large water buffalo, shared feeding by tens of lizards can take place over several days.

**Figure 2:**
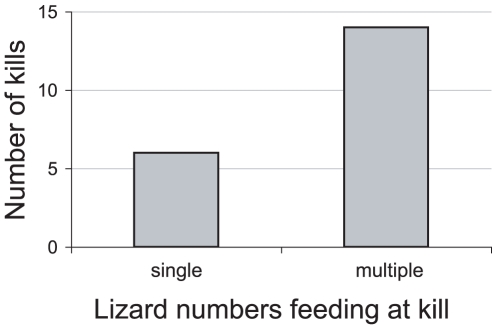
Communal feeding by dragons. Incidence of single versus multiple Komodo dragons feeding on large prey (Timor deer, wild pigs, water buffalo, and a Hawksbill sea turtle). Based on 20 independent observations of Komodo dragon feedings noted during fieldwork activities between 2002–2009.

**Figure 3:**
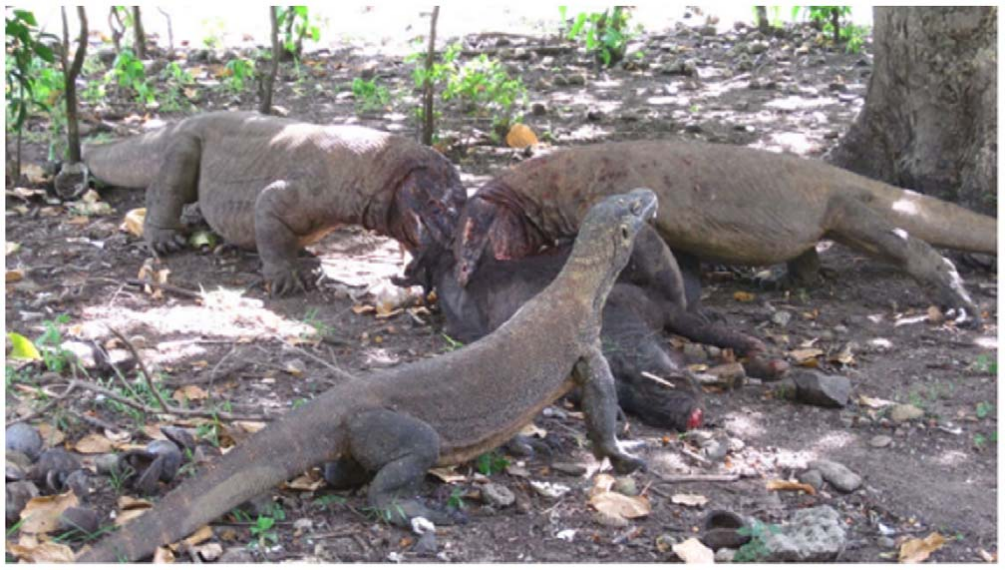
Communal feeding illustrated. Three dragons are feeding on a wild pig. The large prey size of Komodo dragons with overlapping lizard home range generally precludes a single lizard from consuming its large prey alone. Multiple or shared feeding facilitates spread of infectious bacteria between lizard mouths. (Photo by Achmad Ariefiandy.)

In the course of field studies (by TSJ), twenty independent dragon feeding episodes on different large prey were observed: deer (8 observations), buffalo (7), pigs (4) and one sea turtle. Seventy per cent of these kills involved feeding by multiple lizards (Figs. 2, 3). Consumption is preferentially dominated by larger lizards for Timor deer prey, but potentially includes smaller lizards if the prey is sufficiently large (e.g., water buffalo). It is noteworthy that, although a single buffalo carcass experiences enough communal feeding to infect more than a dozen lizards under this model, communal feeding is the norm for various types of large prey and thus will allow the model to operate even if bite-induced infections and subsequent prey death occur uncommonly. Note that the incidence of multiple lizards at a single prey is likely to depend on lizard density and so should be more common on large islands than on small islands–small islands have low dragon densities [7],

#### 3. Infectious oral bacteria in lizard mouths

There are miscellaneous reports of infections from dragon bites. Auffenberg [2] devoted 2–3 pages to the subject, citing a human sepsis death within a week of a large lizard's bite to the person's arm. These observations appear to have motivated Auffenberg's proposal of what we have termed the ‘bacteria as venom’ model. Further, the legend to an illustration of a bite injury on a buffalo's lower leg comments on edema from infection. However, Auffenberg [2] and Komodo National Rangers commented on personally experiencing no infection from bites of moderately small dragons (<60 cm SVL). This suggests that risk of bacterial infection varies among lizards or perhaps with the severity of the bite, possibly dependent on lizard body size. An increasing incidence of sepsis-inducing bacterial with lizard size is expected under the model, given that large lizards dominate communal feeding.

As discussed next, the data on dragon oral bacterial identities and characteristics are consistent with the lizard-lizard epidemic model but, on close inspection, are not detailed enough to provide much resolution. It should be realized that the ‘epidemic’ model, if correct, may apply to as little as a single species in dragon mouths but could also apply to a consortium of several species, no one of which is sufficient to cause an infection in prey. However, since the infectious flora is maintained via an epidemic among lizards, the same infectious bacteria should be found in different (adult) lizards, at least within each physically defined lizard population. Over time, the dominant infectious bacteria may turn over, and which bacterial species prevail(s) will depend on dynamical interactions in the lizard mouth as well as on the effects on prey.

The single published survey of bacteria cultured from wild Komodo dragon mouths observed a diverse bacterial population [3], including many known pathogens. As will be noted below, interpretation of those data is hampered by possible biases in bacterial culturability and by the lack of information about the sizes of individual lizard hosts.

The sampled oral flora from wild lizards included *Staphylococcus aureus*, *Pseudomonas aeruginosa* and other bacteria well known as common opportunistic pathogens capable of infecting and killing a wide array of organisms. However, the Gram-negative bacterium *Pasteurella multocida* is the most noted bacterial pathogen isolated from the dragon mouth. *P. multocida* is a common commensal of the oral cavity in domestic and wild mammals. Upon transmission to humans via animal bites or scratches, *P. multocida* often causes localized cellulitis and even causes septicemia in some cases [8], [9]. The fact that *P. multocida* does not commonly cause purulent infections in humans bitten by their pets might argue against this bacterium fitting the ‘epidemic’ model for dragons, but it would be premature to regard all strains of *P. multocida* (or of any other bacterial species) as equivalent. Furthermore, the bites of dragons may be more conducive to establishing an infection than bites of other types of animals. Indeed, experimental infections of mice with dragon saliva proved lethal from 3 of 5 lizards, and *P. multocida* was the only bacterium isolated from the dying mice.

Nonetheless, *P. multocida* is merely a candidate for this model. Although this survey is suggestive of an infectious oral flora consistent with the ‘epidemic’ model, further work is needed to provide a critical test. The survey is inconclusive for two reasons. (A) The existence of an infectious oral flora may seem to be highly supportive of the model, but an infectious flora is not unique to dragon mouths. Studies on snakes, for example, also find opportunistic pathogens in their mouths [10], [11], and snakes cannot be construed to fit the ‘epidemic’ model. Likewise, the most noted infectious agent in dragon mouths, *P. multocida*, is common in the oral cavity of many mammals, e.g., 70%–90% in cats [12], [13] and 50%–60% in dogs [14], [15]. Absence of an infectious agent in dragon mouths would be fatal to the model, but the collective presence of several agents is compatible with many other models as well as with the ‘epidemic’ model.

(B) The most abundant bacterial species was found in only 14 of the 26 wild dragon mouths tested (an unidentified *Streptococcus* species), and the next most common species was an unidentified *Staphylococcus* observed in 10 mouths [3]. In particular, *P. multocida* was listed as cultured from only 2 wild dragons. At face value, this observation is fatal to the model, at least if most wild dragons in the study were large adults from one population (which is unknown). *P. multocida* is clearly not the only bacterium that is a candidate for the ‘epidemic’ model, but that species also may have been far more common in lizard mouths than detected in this survey. For example, *P. multocida* was cultured from mice inoculated with saliva of 3 lizards, even though cultures from saliva tested positive for only 1 of those lizards. Of three lizards whose saliva was negative for *P. multocida*, all exhibited antibodies against *Pastuerella*.

The methodology used by Montgomery et al. [3], bacterial enrichment under aerobic conditions in a commercial medium without serum or blood, likely drastically underestimated the true abundance and diversity of pathogenic bacteria. Additionally, culture-based methods do not take into account unculturable bacteria, including bacteria in the viable but non-culturable (VBNC) state which may be prevalent in many natural environments [16]. These facts are even relevant to *P. multocida*–which was isolated from some lizards - which often has complex growth requirements and is normally cultured in media containing mammalian blood in a 5% CO_2_ atmosphere. Although the limited incidence of *P. multocida* reported in the Montgomery study was used to argue that this bacterium is not a common commensal of the Komodo dragon [4], a more rigorous molecular-based study of bacterial diversity is needed to answer this question. If it can be shown that no infectious bacterial strain is common to most adult lizards, the ‘epidemic’ model will be refuted. Importantly, the model requires not merely that the same bacterial species be present in different adult lizards, but specifically the same strain, with minimal evolutionary divergence (reflecting a recent common demographic history).

The data reported by the Montgomery study are thus inconclusive in supporting or refuting the epidemic model. They are useful in illustrating both the difficulty of using standard culture methods to assess bacterial communities and in revealing that dragon mouths harbor many bacterial species that could potentially spread as an oral epidemic under the right life history conditions.

#### 4. Bacterial survival and colonization

The R_0_ of an oral bacterium is enhanced by its ability to colonize and persist in the lizard mouth and especially by its endurance in a carcass. As carrion is a common element in the lizard diet, a bacterium that causes prey death and also persists indefinitely after prey death has an excellent chance of introduction into lizard mouths. Even for septic prey that are killed by lizards, a large prey (e.g., water buffalo) may be consumed over several days, and survival in carrion affords the bacterium further opportunities to colonize lizards. *P. multocida*, noted above as a sepsis-inducing bacteria inhabiting at least some lizard mouths, has been shown to survive for weeks in water and carrion [17]–[19]. Bacterial survival in carrion is so extreme that carcass collection to reduce bacterial contamination is the current method used to control *P. multocida* levels in wetlands. The fact that *P. multocida* is known to be a common commensal of mammalian mouths (point 2 above) suggests that it may be predisposed for colonization of lizard mouths. Of course, any bacterium persisting in lizard mouths may evolve and adapt to that environment, so characteristics of the same bacterial species from other environments may not match its characteristics after adaptation to lizard mouths.

## Discussion

There are now three explanations for an infectious flora of lizard mouths, the ‘bacteria as venom’ model, the ‘passive acquisition’ model and the ‘lizard-lizard epidemic’ model. This paper has concentrated on developing and testing the latter. Here, we turn to consider all three.

The ‘bacteria as venom’ model was proposed casually, without specifying many of its properties [2]. We consider it untenable, as did Frye et al. [4]. It requires that lizards with bacteria have higher survival and/or reproduction than lizards lacking infectious oral bacteria. One major problem with the model is that the lizards are apparently born without infectious oral bacteria (based on the virtual absence of those bacteria in captive animals). Thus lizards must first acquire highly infectious oral bacteria and then benefit disproportionately from those bacteria. But then the flora dies out when the lizard dies, rather than being transmitted to offspring. Thus, even if lizards benefit from an infectious oral flora, there is no dynamic or evolutionary process to maintain the bacterial association across generations. A second problem is that any prey escaping an initial attack is often not re-captured by the same lizard. So the benefit of having one's bacteria kill an escaped prey does not typically go to the lizard carrying those bacteria.

**Figure 4:**
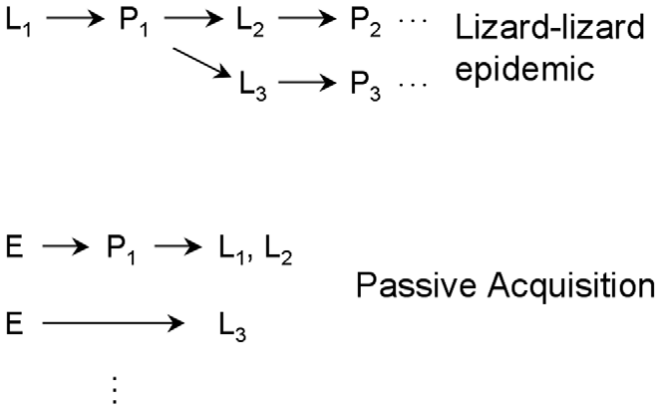
Bacterial-lizard dynamics differ between the Passive Acquisition and Lizard-Lizard Epidemic models. The arrows show the transmission of bacteria that ultimately colonize lizard mouths. In the lizard-lizard epidemic model, bacteria colonize new lizard mouths (L_2_, L_3_) when those lizards eat prey (P_1_) infected from another lizard (L_1_). The prey ultimately eaten by lizards 1 and 2 must be injured during but escape a lizard attack and survive long enough for the infection to develop. In contrast, lizards in the passive acquisition model acquire their oral bacteria either directly from the environment (E, lower arrow) or from prey that acquired bacteria environmentally. There are no chains of transmission between lizards in the latter model.

The two other models remain tenable at this point. There is, however, a strong asymmetry in the ease with which each is refuted. The ‘passive acquisition’ model, also proposed casually, potentially encompasses a wide variety of mechanisms by which the lizards acquire bacteria from their environment (Fig. 4), so identifying ways to falsify it presents major difficulties. Indeed, we expect that the model explains much of the oral flora in most vertebrate species, even most of the oral flora in dragons. That model is most easily tested relative to an alternative that incorporates a clear and specific set of assumptions, such as the ‘epidemic’ model. Here, we showed that the epidemic model survived initial tests based on unique characteristics of lizard life history.

Table 1 lists four possible future tests that were not considered above; those tests will require new data. The most compelling support for the epidemic model will be the direct observation of lizard-to-lizard bacterial transmission via an infected prey, but observations of the same infectious bacterial strain among adult lizards (and only in lizards) would certainly be suggestive. Such evidence–pro or con - should be obtainable with molecular population genetics methods of bacterial samples from lizard mouths. These are not the only tests that might be applied, but they are some of the more obvious ones to consider in the next round.

**Table 1:** Further tests to refute the lizard-lizard epidemic model (LLE) and passive acquisition (PA) models.

Possible Observation	Model refuted
Infectious bacterial strain is found only in lizard mouths and bitten prey	PA
Infectious bacterial strain found in young lizards	LLE
Most adult lizards do not share the same infectious strain	LLE
Bacterial strain tracked from lizard through prey to other lizards	PA

### Other systems

The lizard-lizard epidemic model proposed here can operate in other predators, including mammals. Auffenberg [2] discussed the remote possibility that lynx predation on young caribou involved infectious oral bacteria. A priori, one might expect that the ecological characteristics most prone to fit an infectious spread of toxic oral bacteria would be a high density of predators whose prey were as large as or larger than the predator (e.g., canids hunting large mammals), facilitating prey escape and enhancing the rate of subsequent encounter by other predators. However, there are two properties of dragons that differ from mammalian predators and may predispose lizards toward this model. First, as large reptiles, dragons are no doubt long-lived. Since the bacteria do not kill the lizards, a long intrinsic lizard lifespan increases the R0 of the oral infection, provided that the bacteria can persist in the lizard mouth. Second, lizard physiology differs from mammal (prey) physiology. When first acquiring a sepsis-inducing bacterium, the lizard physiology may help protect them from developing sepsis from a bacterium that causes a lethal sepsis in their mammalian host. A mammalian predator of a mammalian prey may be prone to die of the infection that kills its prey.

## Methods

### Lizard natural history

Since 2002, TSJ has been involved in field ecology studies of *V. komodoensis* at ten sites on four islands in Komodo National Park, Indonesia [1], [7]. These studies have assessed ecological interactions among Komodo dragons and their prey. Each year, approximately 140 days is allocated to field work that includes mark-recapture of dragons, ungulate distance sampling, dragon nest surveys, and indirect abundance surveys for prey. This field work also provides the opportunity to incidentally observe interactions between Komodo dragons and their prey (deer and pigs, in this case), which are reported here as rates of injured prey escape. Some observations were also collected during student studies that followed adult dragons via radio telemetry and were able to directly observe prey attacks [20].

### A formal lizard-lizard epidemic model

To test whether our intuition about the process of the infectious spread of an oral flora is accurate, we used a quantitative model. This model is necessarily elementary, both because we cannot hope to capture the complexities of lizard ecology and population structure in any manageable set of equations and because there is no information with which to parameterize the model.

The model addresses whether an infectious oral flora can spread when rare in a population of uninfected lizards. This model does not address the full dynamics, but restricting the analysis to invasion dynamics simplifies the analysis, because it means that we can confine the dynamics to just two groups: infected lizards and infected prey. The much larger populations of uninfected lizards and uninfected prey can be considered constant as long as the infected types are rare, and interactions between infected prey and infected lizards can be ignored; the time scale considered is short enough to neglect lizard death as a source of change in the abundance of infected lizards.

In setting up the equations, we note that

- Numbers of infected lizards change in two ways: a reduction when previously infected lizards lose their oral flora; a gain when uninfected lizards eat infected prey. This gain is higher with shared feeding (a function of prey size), captured with the parameter 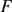.
- Numbers of infected prey change in 3 ways: a gain when infected lizards bite uninfected prey that escape the initial attack; a loss when infected prey die without being eaten; a loss when infected prey are eaten by one or more lizards.

Equations for the changes in abundances of both types are thus
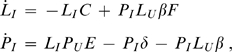
where a superior dot indicates a rate of change, and the notation is as follows:


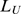
density of uninfected lizards


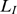
 density of infected lizards


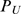
 density of uninfected prey


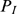
 density of infected prey


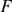
 rate constant at which uninfected lizards feed on killed, infected prey and become colonized (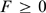)


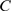
 rate constant at which an infected lizard mouth is cleared to a non-infectious state


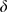
 rate constant at which an infected prey dies without being eaten


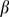
 rate constant at which an infected prey is killed by an uninfected lizard


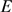
 rate constant at which an uninfected prey is attacked, fully escapes the attacking lizard, and develops sepsis from the lizard's bacteria

Note that some parameters encompass multiple steps. For example, 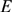 encompasses attack on a prey, escape, and infection of the prey. The parameter 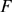, which appears only in the equation for 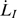, accounts for the possibility that multiple lizards may feed on and be colonized from a single infected prey (communal feeding). A more accurate model would cap the life span of infected prey (they will all be dead from the infection after a few days, whereas the model has them die at a constant rate), would also increase the probability of lizard predation of infected prey with days post infection, and would allow dead, infected prey to be consumed. Such complexity is beyond the scope of this simple analysis and would not lend itself to analytical tractability.

The characteristic equation for this system is 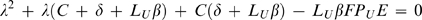



The infection spreads if 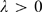, which requires 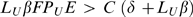



Several properties of this result agree with intuition. First, high rates of (i) communal feeding and colonization of lizard mouths (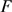), (ii) of prey escaping and becoming infected (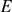), and (iii) of infected prey being subsequently killed by uninfected lizards (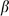) all help the spread of the oral infection. Second, reducing the clearance rate of oral infections (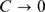) and reducing the rate at which infected prey die without being consumed by lizards (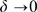) also contribute to spread. These points are obvious. Third, because the model assumes mass action, it is understandable that high densities of uninfected prey and of uninfected lizards both increase the rates at which infections spread. Less obvious is how some of the terms interact. For example, reducing 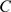 to 0 ensures that the infection will spread (although this outcome depends on the assumption that lizards do not die), whereas reducing 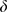 to 0 does not ensure spread.

The model enables us to present a verbal description about the type of life history needed to explain an infectious oral flora as an infectious ‘disease’ of lizards (as per Results). Ideally, we would hope to parameterize the model with data from dragons and prey to see if the model is quantitatively plausible, but that is not currently possible because so little is known about the natural history of these lizards and their oral flora.

This analysis addresses only the spread of the oral flora when rare. A complete model would provide dynamics—how common the infection was among lizards at dynamical equilibrium, oscillations, and turnover rates. Furthermore, the model has omitted age structure of the lizards: an infectious oral flora should be more common in old animals than young ones, because (i) it must be acquired after birth, (ii) young dragons do not eat large prey, and (iii) even medium-sized dragons are less inclined to feed communally than large ones due to size-assortative competition for prey resources. There are thus obvious embellishments to include as data accumulate.
